# Servant Girls

**Published:** 1879-02

**Authors:** 


### SERVANT GIRLS.

A FAIR correspondent from one of the most beautiful towns of interior Pennsylvania, writes:

"I know of no place where ladies work harder and are so much enslaved by the hirelings of the kitchen, as in this quiet town of three thousand inhabitants. Six weeks ago I hired a girl, who recommended herself as being very capable of doing work and exceedingly fond of children. I soon found that her capabilities consisted mainly in washing, starching, and ironing her own dresses, of which she never had less than three, with half a dozen collars, in the weekly wash. The second day after she came to me, she walked into my room to use my hair-brush. I looked at her with astonishment. She next went to the wash-stand. 'Mary, there is a basin, towel, soap, and glass in your own room; these articles I do not use in common with any one.' 'Yes, but I like this soap of yours so much,' replied the 'capable' Mary.

"The first sabbath I allowed her to attend church, leaving at noon, to return before supper-time. At ten o'clock she had not come home, so I locked up the house and retired. An hour and a half later there were voices at the door, asking admittance, but they were not heeded. Next morning, about seven, my 'capable' Mary came in, with many reasons for not returning at the specified time. She always came home before night, after that. But she would not get up early enough in the morning. It was necessary for me to call her, and even then, seven o'clock usually found her in bed. This was intolerable, and I dismissed her. She is now in a minister's family, whose wife says, that 'Mary is poor help; she does very little; we must call her two or three times every morning—but what ca[illegible] we do, we are so dependent on our servant girls?'"

From this it would seem that there is rather more trouble in being suited as to help, in the rural districts, than in the city; but whether in city or country, it is not to be denied, that much of the worthlessness of our servants depends on the incompetency of their mistresses. The chief defects are a want of patience, firmness, and due consideration of their ignorance and infirmities. The best servants are those who are sternly held up to the fullest performance of their duty. But, while we do this, we should be considerate in our requirements, courteous in our bearing towards them, and prompt in our payments.

### *CONSTIPATION.*

It is doubtful if consumption numbers as many victims as are stricken down by the various diseases that result from habitual constipation. True consumption is an inherited disease. It may remain always dormant, but when aroused to action, decay commences at a point circumscribed, and gradually extends—unless arrested—until so much of the lungs become involved that vital action ceases. The evils of constipation result from inattention to the calls of nature, and usually commence with children whose habits are not closely looked to by their parents. The processes of nature are always active while life lasts. When effete matter is retained a moment beyond the time its expulsion is demanded, the system commences its efforts to get rid of it. When the natural egress is checked, the absorbents carry the more fluid portions of the poisonous mass into the circulation, and it becomes diffused throughout the body. The more solid or clay like portion is forced into the lower rectum where it becomes firmly impacted, thus cutting off the circulation in the small blood vessels, causing painful engorgement known as piles and hemorrhoids. A continuance of these troubles often results in fissure, fistula, or cancer. The trouble is seldom confined here. As a result of the blood poisoning we almost invariably find more or less dyspepsia, with decided derangement of the functions of the heart, liver, and kidneys, accompanied by headache and nervous debility, often verging on paralysis.

Persons of sedentary habits are most subject to constipation and its sequences. Before the plan of treatment devised by M. Nelaton was introduced, habitual constipation was a disease of far more serious import than at the present time. All physicians admit that cathartics afford only temporary relief, and catharsis cannot be induced except by stimulating the bowels with irritants, and as this irritative process gradually weakens their peristaltic action, the relief from them steadily diminishes until disease of some vital organ sets in, and terminates life. Relief from injections is just as pernicious, with the added danger of strictures of the rectum. With these facts in view, M. Nelaton commenced his investigations, which resulted in proving beyond question that in all uncomplicated cases of constipation, the obstruction exists solely in the lower portion of the rectum, and within reach of local remedies. For the radical cure of the various diseased conditions, he invented "Nelaton's Suppositories." It is said they succeeded beyond his own expectations. But in old and severe cases their use must be persisted in until the cure is complete.

---

**Nelaton's Suppositories are sold by druggists at $1 00 a box. Hall & Ruckel, wholesale druggists, New York, are agents for the United States, and send them free by mail on receipt of price.**

